# Identification of an Immune-Related Gene Signature Associated with Prognosis and Tumor Microenvironment in Esophageal Cancer

**DOI:** 10.1155/2022/7413535

**Published:** 2022-12-23

**Authors:** Chunzhen Li, Weizheng Zhou, Ji Zhu, Qi Shen, Guangjie Wang, Ling Chen, Tiejun Zhao

**Affiliations:** ^1^School of Basic Medicine, Naval Medical University, Shanghai 200433, China; ^2^Department of Thoracic Surgery, The First Affiliated Hospital of Naval Medical University, Shanghai 200433, China

## Abstract

**Background:**

Esophageal cancer (EC) is a common malignant tumor of the digestive system with high mortality and morbidity. Current evidence suggests that immune cells and molecules regulate the initiation and progression of EC. Accordingly, it is necessary to identify immune-related genes (IRGs) affecting the biological behaviors and microenvironmental characteristics of EC.

**Methods:**

Bioinformatics methods, including differential expression analysis, Cox regression, and immune infiltration prediction, were conducted using R software to analyze the Gene Expression Omnibus (GEO) dataset. The Cancer Genome Atlas (TCGA) cohort was used to validate the prognostic signature. Patients were stratified into high- and low-risk groups for further analyses, including functional enrichment, immune infiltration, checkpoint relevance, clinicopathological characteristics, and therapeutic sensitivity analyses.

**Results:**

A prognostic signature was established based on 21 IRGs (S100A7, S100A7A, LCN1, CR2, STAT4, GAST, ANGPTL5, TRAV39, F2RL2, PGLYRP3, KLRD1, TRIM36, PDGFA, SLPI, PCSK2, APLN, TICAM1, ITPR3, MAPK9, GATA4, and PLAU). Compared with high-risk patients, better overall survival rates and clinicopathological characteristics were found in low-risk patients. The areas under the curve of the two cohorts were 0.885 and 0.718, respectively. Higher proportions of resting CD4^+^ memory T lymphocytes, M2 macrophages, and resting dendritic cells and lower proportions of follicular helper T lymphocytes, plasma cells, and neutrophils were found in the high-risk tumors. Moreover, the high-risk group showed higher expression of CD44 and TNFSF4, lower expression of PDCD1 and CD40, and higher TIDE scores, suggesting they may respond poorly to immunotherapy. High-risk patients responded better to chemotherapeutic agents such as docetaxel, doxorubicin, and gemcitabine. Furthermore, IRGs associated with tumor progression, including PDGFA, ITPR3, SLPI, TICAM1, and GATA4, were identified.

**Conclusion:**

Our immune-related signature yielded reliable value in evaluating the prognosis, microenvironmental characteristics, and therapeutic sensitivity of EC and may help with the precise treatment of this patient population.

## 1. Introduction

According to statistics from 2020, esophageal cancer (EC) has the seventh-highest morbidity and sixth-highest mortality among all cancers [1]. Although surgical resection remains a mainstay of curative treatment for esophageal cancer, the overall survival of patients is still not satisfactory [2]. Therefore, it is imperative to identify new therapeutic and prognostic targets.

In recent years, immunotherapy has gained plenty of attention and is regarded as a promising treatment. Immunotherapy focuses on activating the sufficient response of the body and killing tumor cells by the immune system [3]. Immune checkpoint blockade (ICB) therapy is one of the most promising aspects of immunotherapy, which has been used to treat lung cancer, malignant melanoma, etc. [4, 5]. An increasing body of evidence suggests that ICB drugs, such as nivolumab and pembrolizumab, are optimal second-line treatment strategies for esophageal cancer [6–8]. Nonetheless, only a small proportion of patients can be effectively responsive and benefit from current immunotherapy [9]. The role of the tumor microenvironment (TME) cannot be ignored since multiple microenvironment components, such as immune cells and stromal cells and their signals, reportedly influence the efficacy of immunotherapy. For example, tumor-associated macrophages (TAMs) and regulatory T cells (Tregs) are involved in shaping the suppressive immune microenvironment, which in turn promotes tumor immune evasion and immunotherapeutic resistance [10–12]. The potential of eosinophils as therapeutic targets for cancer has also been revealed, with their proven direct or indirect interactions with tumor cells and other lymphocytes [13]. Therefore, researchers seek to fully exploit the prognostic value and therapeutic potential of immune cells and immune-related molecules. For instance, Xu et al. screened the molecules most associated with eosinophils using a weighted correlation network analysis (WGCNA) and comprehensively investigated the value of this signature in indicating prognosis and therapeutic preference in bladder urothelial cancer [14]. And, immune-related biomarkers have been reported for the prediction of survival risk and clinical response in patients with liver, breast, and colon cancers [15–17]. Accordingly, it is necessary to determine the characteristics of the immune microenvironment of EC and find reliable prognostic indicators to provide new ideas for individualized immunotherapy.

In this study, an immune-related signature that could be used for the prognosis assessment of patients with EC was established and validated using two independent cohorts obtained from public databases. Overall survival outcomes, clinical stage, immune microenvironment, and therapeutic sensitivity differed significantly between high- and low-risk patients classified by this immune-related signature. Moreover, five hub IRGs associated with the progression of EC and their relevance to immune cell infiltration were identified. The immune-related signature yielded a reliable prognostic performance, providing the foothold for individualized therapy of patients with EC.

## 2. Materials and Methods

### 2.1. Data Sources

We obtained the microarray profiling dataset GSE53624 from the Gene Expression Omnibus (GEO) database (https://www.ncbi.nlm.nih.gov/geo/), which includes data from 119 pairs of EC tissues and matching nontumor tissues and their corresponding clinical data. The list of IRGs was obtained from the Immunology Database and Analysis Portal (ImmPort) database [18]. The validation cohort of EC was downloaded from The Cancer Genome Atlas database (TCGA, https://portal.gdc.cancer.gov). After excluding patients lacking clinical information or with inadequate follow-up time (less than 180 days), 111 patients from the GEO cohort and 99 patients from the TCGA cohort were finally included in the study.

### 2.2. Identification of Differentially Expressed Prognostic IRGs

“Limma” (R software package) was used to identify the differentially expressed genes and IRGs [19]. The cutoff values applied were as follows: adj. *P* value <0.05 and log2 |FC| > 1. Volcano plots and heatmaps were generated to visualize the expression of DEGs using the R packages “ggplot2” and “pheatmap.” For functional analysis, Gene Ontology (GO) and Kyoto Encyclopedia of Genes and Genomes (KEGG) pathway enrichment analyses were conducted using the R package “clusterProfiler” [20, 21]. After merging the expression data with corresponding clinical information, prognosis-related IRGs were obtained by performing univariate and multivariate Cox regression analyses.

### 2.3. Establishment of the Prognostic Risk Signature

The prognostic signature of the patient cohort was constructed according to risk scores calculated using this formula:

(1) $$\begin{array}{c} \text{Risk score}=\overset{n}{\underset{i=1}{\sum }}\text{c}\text{oef }\left(\text{IRGi}\right)*\text{expr }\left(\text{IRGi}\right). \end{array}$$

The “coef (IRGi)” represents the regression coefficient of the IRG, which was obtained from Cox analysis. The “expr (IRGi)” is the expression level of prognosis-related IRG [22]. Using this risk signature, patients were stratified into two groups (low- and high-risk groups) with the median value of the risk scores as the cutoff.

### 2.4. Prognostic Performance Evaluation of the Immune-Related Signature

To evaluate the reliability and the predictive effect of the signature, Kaplan-Meier (K-M) analysis was adopted to assess the survival status using the R package “Survival.” The ROC curve was generated to assess the accuracy of the signature using “survivalROC.” The independence of the risk signature from other clinical variables was assessed by univariate and multivariate independent prognostic analyses. To dissect the correlation of the immune-related signature with clinicopathological characteristics, the AJCC (American Joint Committee on Cancer) tumor stage of patients in different risk groups was analyzed and compared.

### 2.5. Construction of the Predictive Nomogram

To predict the 1-, 3-, and 5-year overall survival of patients more quantitatively, we included clinical factors with independent prognostic effects along with risk scores to construct the nomogram using the “rms” package.

### 2.6. Gene Set Enrichment Analysis (GSEA)

To distinguish the functional phenotypes between groups, GSEA was conducted [23]. The “gmt” files for KEGG and GO gene sets were downloaded from the GSEA website (http://www.gsea-msigdb.org/). The top 5 enriched terms or pathways in each group were selected and visualized.

### 2.7. Correlation of the Signature with Tumor Immune Microenvironment

Immune infiltration between groups with different risks was analyzed by running the algorithm “CIBERSORT,” which could show the abundance of 22 different types of immune cells distributed in each sample [24]. Correlations between the risk score and the abundance of immune cells were investigated. We further explored the difference in checkpoint expression between the two risk groups and the correlation of risk scores with the expression of immune checkpoints using the R package “corrplot.” In addition, patients were grouped according to differential immune checkpoint expression and risk scores to further demonstrate survival differences.

### 2.8. Exploration of IRGs Associated with Tumor Progression

The expression of IRGs in different clinicopathological subgroups was also shown to discover IRGs associated with tumor progression. And, we used the Tumor IMmune Estimation Resource (TIMER) database, an online tool for analyzing tumor-infiltrating immune cells based on TCGA datasets, to visualize the effect of key IRG expression and its copy number variation (CNV) on 6 tumor-infiltrating immune cell types [25]. The copy number variation data of ESCA samples were retrieved from the UCSC Xena database (http://xena.ucsc.edu/). The “RCircos” package was applied to visualize the figure [26].

### 2.9. Therapeutic Response Prediction

The Tumor Immune Dysfunction and Exclusion (TIDE) scores were adopted to quantify the potential benefit of immunotherapy, and lower TIDE scores are associated with a better response to immunotherapy [27]. Moreover, eight chemotherapeutic drugs (docetaxel, doxorubicin, gemcitabine, imatinib, sorafenib, roscovitine, lenalidomide, and rapamycin) were selected for the chemotherapeutic response prediction. Half-maximal inhibitory concentration (IC50) of these drugs was calculated by “pRRophetic” to compare the differences between risk groups [28, 29].

### 2.10. Statistical Analysis

We used R software, a practical tool that contains multiple packages, to perform statistical analysis. Student's *t*-test or Wilcoxon's test was applied to evaluate between-group comparisons of continuous variables. Group comparisons of categorical variables were performed using *χ*^2^ or Fisher's exact tests. *P* < 0.05 was regarded as statistically significant if not specifically stated. After the statistical analysis, multiple R packages, including “ggplot2,” “ggpubr,” “pheatmap,” “survival,” and “ggExtra” were used to visualize the results.

## 3. Results

### 3.1. Identification of Differentially Expressed IRGs (DEIRGs) and Prognosis-Related IRGs

**Figure 1:**
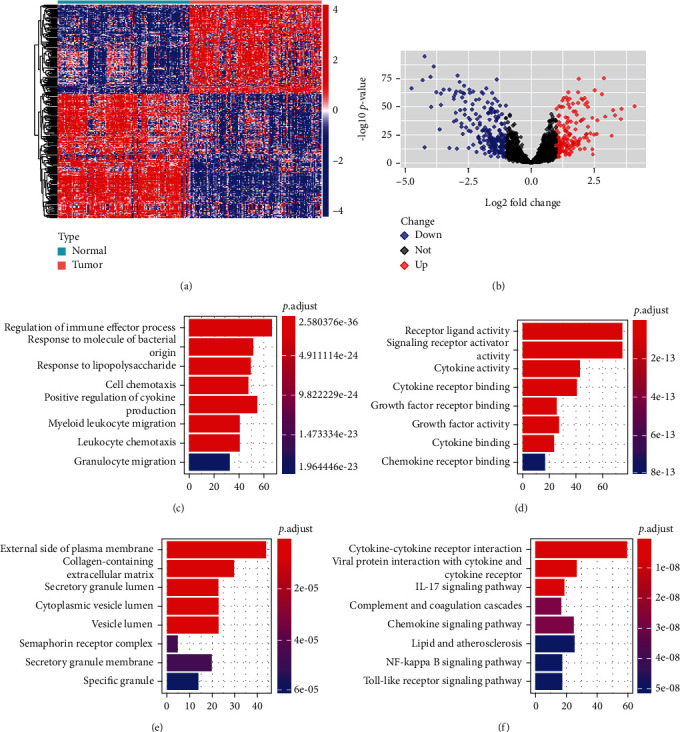
Identification of DEIRGs and functional enrichment analysis. Heatmap (a) and volcano map (b) of DEIRGs. The blue-to-red spectrum in the heatmap indicates the low to high expression of genes. In the volcano map, upregulated genes and downregulated genes are indicated by red and blue dots, respectively. Significant GO terms of molecular function (c), biological processes (d), and cellular components (e). (f) The top 8 enriched KEGG pathways.

We obtained 4059 differentially expressed genes (DEGs) in EC tissues compared to nontumor tissues. The intersection of DEGs with immune-related genes yielded differentially expressed immune-related genes (DEIRGs) consisting of 150 upregulated and 209 downregulated genes (Figures 1(a) and 1(b)). Then, the GO and KEGG pathway enrichment analyses were conducted. Significant GO modules, including molecular function (MF), biological processes (BP), and cellular components (CC), are shown in Figures 1(c)–1(e). The receptor-ligand activity, the regulation of immune effector response, and the external side of the plasma membrane were the top enriched terms in MF, BP, and CC, respectively (Figures 1(c)–1(e)). Moreover, cytokine-cytokine receptor interactions and other inflammatory processes were the most significantly enriched KEGG pathways (Figure 1(f)).

Based on 359 DEIRGs, we performed univariate and multivariate Cox regression analyses to screen IRGs associated with survival outcomes. Then, a list of 21 prognosis-related DEIRGs for subsequent signature construction was obtained (Table 1).

### 3.2. Construction and Verification of the Prognostic Signature

Twenty-one prognostic IRGs and their coefficients were screened and calculated for subsequent signature construction. The individual risk scores were determined using the formula: Risk score = (0.2107 × S100A7 level) − (0.2760 × S100A7A level) + (0.5854 × LCN1 level) − (0.3311 × CR2 level) + (1.0717 × STAT1 level) − (0.3989 × GAST level) + (0.4201 × ANGPTL5 level) − (1.4978 × TRAV39 level) + (0.7696 × F2RL2 level) + (0.3421 × PGLYRP3 level) − (0.8977 × KLRD1 level) + (0.7635 × TRIM36 level) − (0.5720 × PDGFA level) − (0.4375 × SLPI level) + (0.3789 × PCSK2) + (0.3202 × APLN level) − (1.2788 × TICAM1 level) + (0.7387 × ITPR3 level) + (1.0494 × MAPK9 level) + (0.4076 × GATA4 level) + (0.8366 × PLAU level).

**Figure 2:**
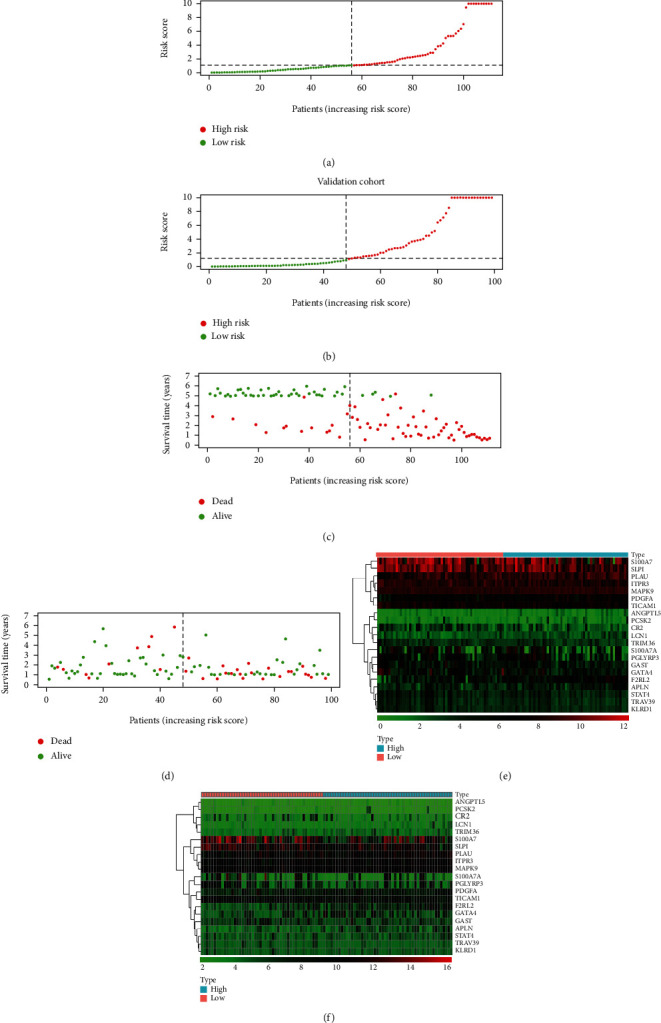
Construction of the signature. (a, b) Distribution of risk scores and group division in two patient cohorts. (c, d) Overall survival status between groups. (e, f) Heatmap of IRGs involved in the signature. Results of (a, c, e) are based on the GEO (training) cohort; results of (b, d, f) are based on the TCGA (validation) cohort.

**Figure 3:**
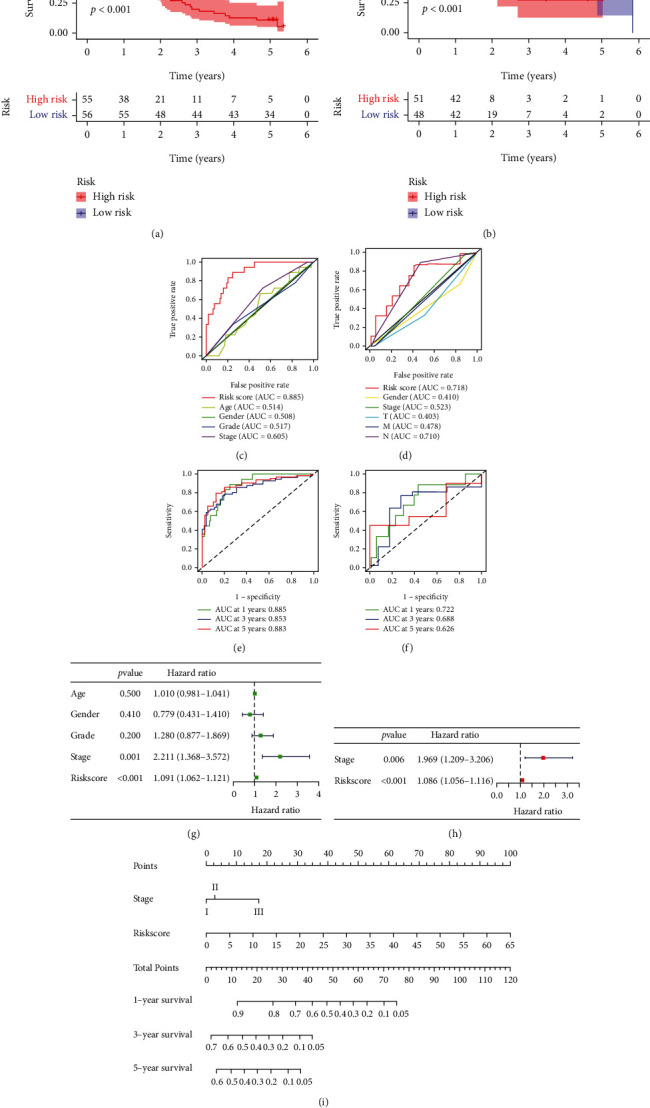
Evaluating the prognostic signature. (a, b) Kaplan–Meier analysis of the two cohorts showed that high-risk patients exhibited a shorter overall survival time (*P* < 0.001). ROC curves of the signature in the GEO (c, e) and TCGA cohorts (d, f). (g, h) The signature was an independent indicator of patient prognosis. (i) The prognostic nomogram for predicting survival outcomes.

High-risk and low-risk groups of patients were then determined based on the median risk score (Figures 2(a) and 2(b)).  Figure 2(c) shows the survival status of patients in the GEO cohort, while the survival status of patients TCGA cohort was shown in Figure 2(d). The differential expression of prognostic genes between groups was shown in heatmaps (Figures 2(e) and 2(f)). As time went on, the survival rate of high-risk patients in the GEO cohort (Figure 3(a)) and the TCGA cohort (Figure 3(b)) was markedly lower than that of the low-risk patients (*P* < 0.001).

### 3.3. Evaluation of the Signature and Construction of the Predictive Nomogram

To assess the predictive efficacy of the immune-related signature, time-dependent ROC curves and multi-indicator ROC curves were plotted. We then calculated the AUC of the ROC curve of the GEO cohort (0.885) and the TCGA cohort (0.718) (Figures 3(c) and 3(d)). The 1-, 3- and 5-year ROC curves also indicated a satisfactory performance of the signature (Figures 3(e) and 3(f)). As indicated by the Cox regression analyses (Figures 3(g) and 3(h)), the risk score could act as an independent prognostic factor (HR = 1.066, 95% CI 1.056–1.116, *P* < 0.001). Overall, these results indicated the reliability of our risk signature for suggesting patient prognosis. Therefore, a nomogram was constructed incorporating the risk score and another independent prognostic factor, tumor stage (Figure 3(i)).

### 3.4. Gene Set Enrichment Analysis (GSEA)

**Figure 4:**
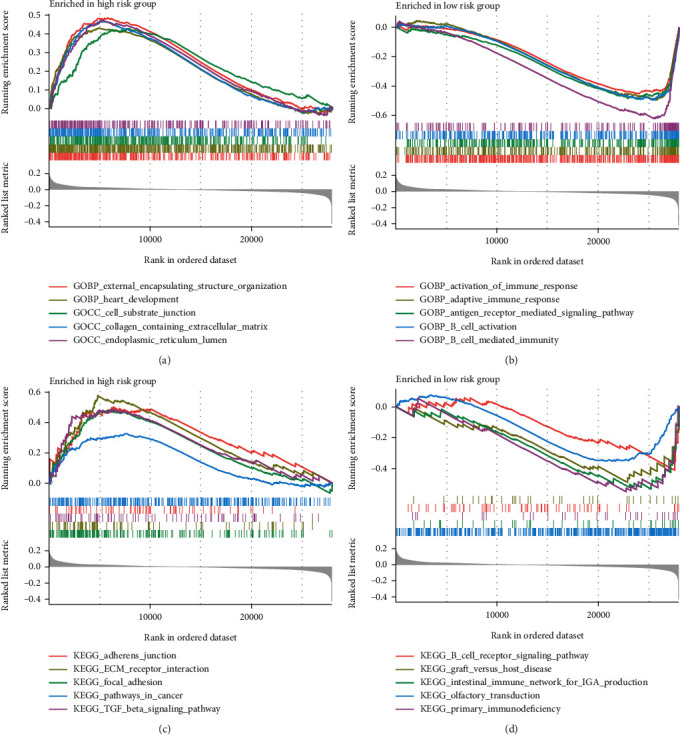
GSEA results. (a, b) The top 5 enriched GO terms in the high-risk and low-risk groups, respectively. (c, d) The top 5 enriched KEGG pathways in the high-risk and low-risk groups, respectively.

Moreover, GSEA showed that genes were significantly enriched in GO terms such as external encapsulation structure organization and cell-substrate function in the high-risk group (Figure 4(a)). Significantly enriched pathways in high-risk patients included adherens junction, pathways in cancer, and TGF-*β* signaling pathways (Figure 4(c)). In low-risk patients, B cell-mediated immunity, activation of immune response, and B cell receptor signaling were significantly enriched, suggesting a stronger correlation between the risk signature and immune bioprocess, especially B cell immunity (Figures 4(b) and 4(d)).

### 3.5. Characteristics of Tumor Immune Microenvironment

**Figure 5:**
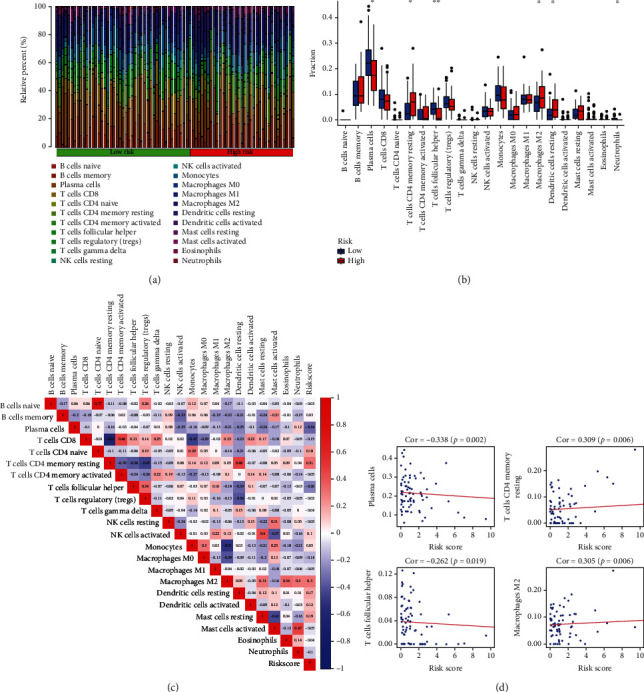
Immune cell infiltration. (a) Immune cell infiltration status in tumors. (b) The difference in tumor-infiltrating immune cells between groups. (c) Correlation of the risk scores with immune cell infiltration. In the heatmap, the blue-to-red spectrum indicates negative to positive correlation. (d) Scatter plots showed the correlation of risk scores with four different immune cell subtypes. ^∗^*P* < 0.05; ^∗∗^*P* < 0.01.

Next, we sought to explore the immune infiltration in tumors with different risks via analysis of immune cell subtype abundance by CIBERSORT (Figure 5(a)). The fraction of subtypes showed significant differences between groups, including follicular helper T cells, CD4^+^ memory resting T cells, M2 macrophages, neutrophils, resting dendritic cells, and plasma cells (Figure 5(b)). The infiltration of M2 macrophages and CD4^+^ memory resting T cells positively correlated with the risk scores (Figures 5(c) and 5(d)). The infiltration of follicular helper T cells and plasma cells correlated negatively with the risk scores (Figures 5(c) and 5(d)).

**Figure 6:**
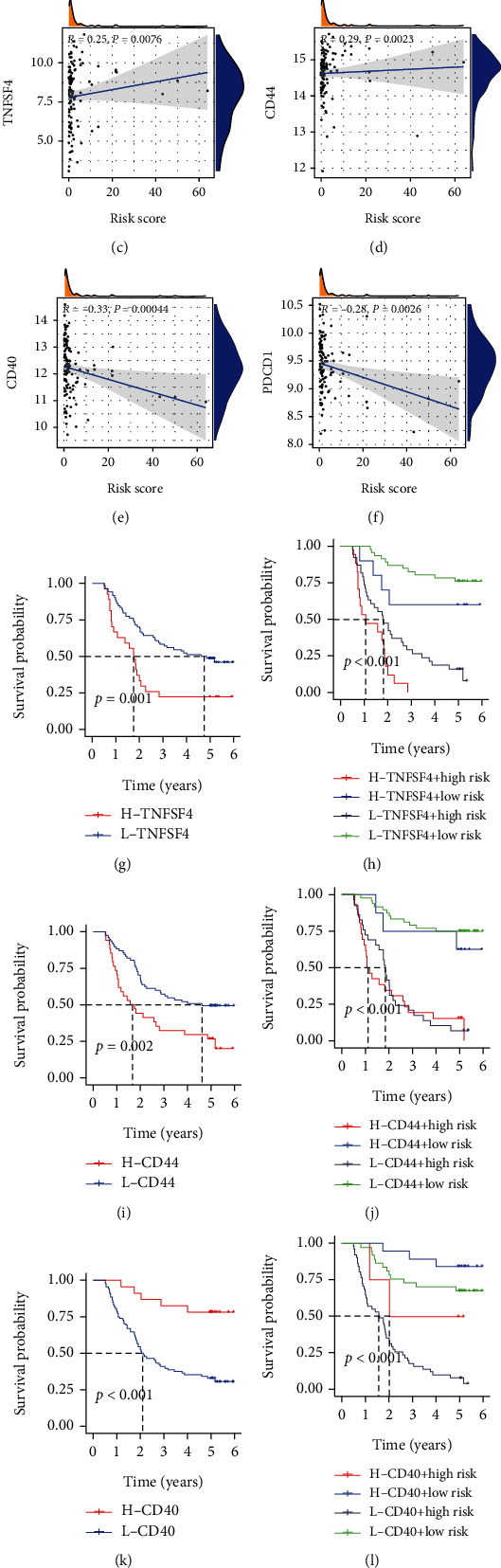
Immune checkpoint expression. (a) Correlation of risk scores with the expression of 22 immune checkpoints. (b) Differential immune checkpoints between groups. (c–f) Scatter plots show the correlation of risk scores with the expression of TNFSF4 (c), CD44 (d), CD40 (e), and PDCD1 (f). Survival analysis based on the signature combined with the expression of TNFSF4 (g, h), CD44 (i, j), CD40 (k, l), and PDCD1 (m, n). ^∗^*P* < 0.05; ^∗∗^*P* < 0.01.

Immune checkpoints play essential roles in regulating the immune response and TME. Accordingly, we assessed the correlation between the risk score and the expression of 22 common immune checkpoint molecules; TNFRSF4, PDCD1, PDCD1LG2, HAVCR2, CTLA4, and CD40 strongly correlated with the risk score (Figure 6(a)). Four checkpoints, TNFSF4, PDCD1, CD40, and CD44, were differentially expressed between groups (Figure 6(b)). In the high-risk group, the expression levels of CD44 (*P* < 0.05) and TNFSF4 (*P* < 0.01) were higher. In contrast, the expression levels of CD40 and PDCD1 were downregulated (*P* < 0.05). With increased risk scores, the expression levels of TNFSF4 and CD44 were upregulated (Figures 6(c) and 6(d)). Lower levels of CD40 and PDCD1 were associated with higher risk scores (Figures 6(e) and 6(f)).

We also explored the effect of risk scores and checkpoint expression on patient survival. Patients with higher expression of TNFSF4 (*P* = 0.001) or CD44 (*P* = 0.002) had poor survival (Figures 6(g) and 6(i)). Among high-risk patients, high TNFSF4 or CD44 was associated with the worst overall survival, while low-risk patients with low TNFSF4 or CD44 experienced the best survival (*P* < 0.001; Figures 6(h) and 6(j)). On the contrary, higher expression of CD40 or PDCD1 correlated with a favorable survival (*P* < 0.001; Figures 6(k) and 6(m)). We also found that low-risk patients with elevated expression of CD40 or PDCD1 experienced the best overall survival (*P* < 0.001; Figures 6(l) and 6(n)).

### 3.6. Clinical Relevance of the Signature and Exploration of IRGs Associated with Tumor Progression

**Figure 7:**
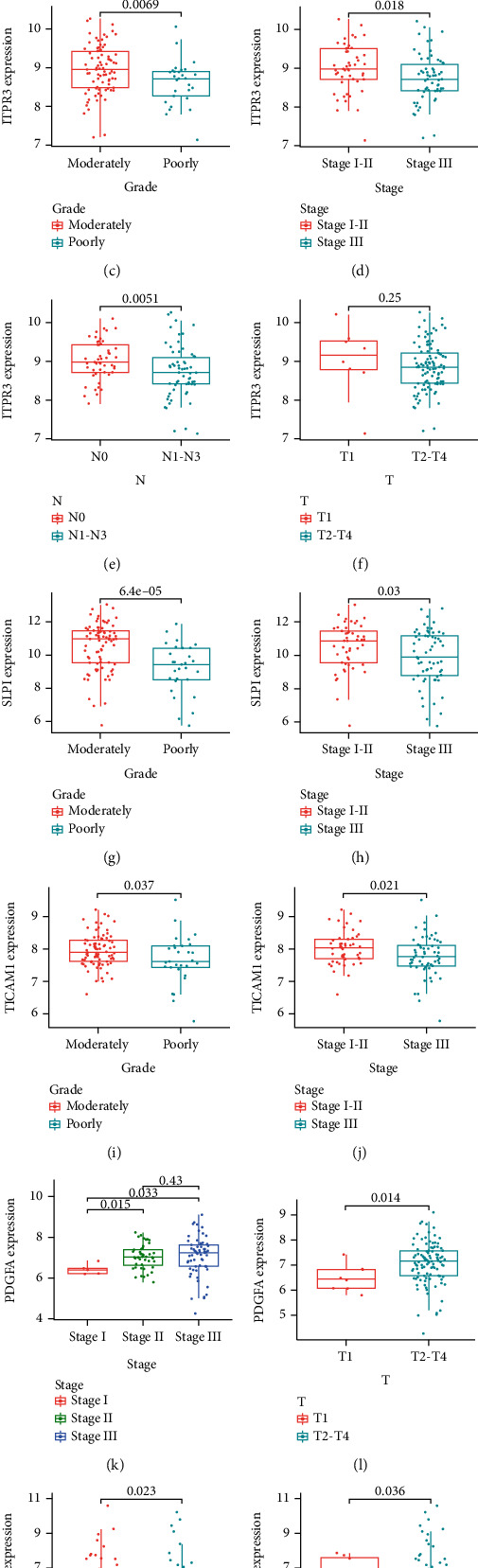
Clinical relevance of the signature. (a, b) Heatmap showed that the prognostic signature correlated with the stage and N-stage. (c–n) Correlation between clinical variables and expression of IRGs; (c–f) ITPR3; (g, h) SLPI; (i, j) TICAM1; (k, l) PDGFA; (m, n) GATA4.

The correlation between risk scores and clinicopathological parameters (i.e., age, gender, tumor grade, and TNM stage) was analyzed. The differences in tumor stage (*P* = 0.041) and N-stage (*P* = 0.014) in different groups were statistically significant (Figures 7(a) and 7(b)).

In addition, the gene expression levels of patients with different clinicopathological features were compared. With increased clinical grade, stage, and N-stage of the tumor, ITPR3 expression decreased (*P* < 0.05, Figures 7(c)–7(e)). In contrast, no significant difference in ITPR3 expression in patients with different T stages was observed (Figure 7(f)). Expression levels of SLP1 and TICAM1 also decreased with advanced clinical grade and tumor stage (*P* < 0.05, Figures 7(g)–7(j)). Notably, the expression of PDGFA in groups with advanced clinical stage and T stage was higher than those with moderate-stage disease (*P* < 0.05, Figures 7(k) and 7(l)). In addition, the decrease of GATA4 expression with more advanced disease stage and T stage of the tumor was statistically significant (*P* < 0.05, Figures 7(m) and 7(n)).

**Figure 8:**
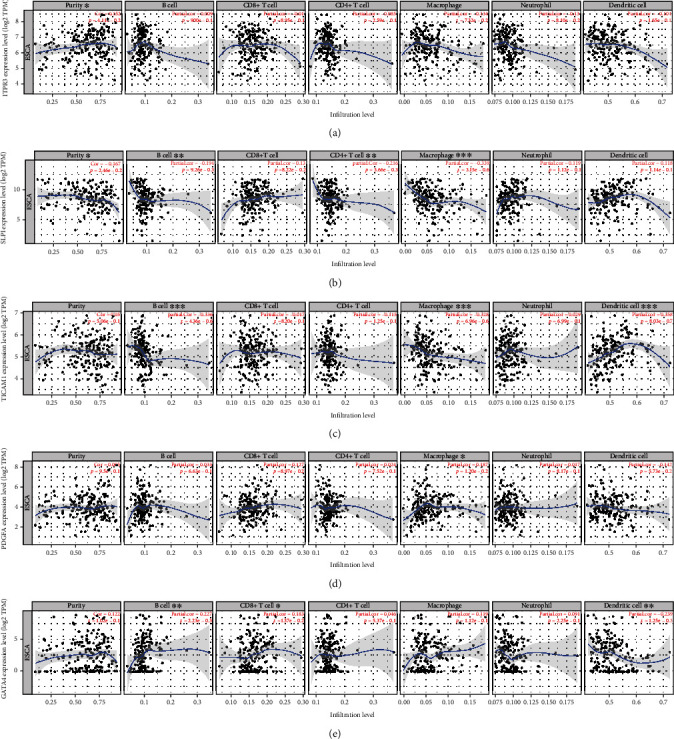
Correlation of IRG expression with the abundance of tumor-infiltrating immune cells. (a) ITPR3; (b) SLPI; (c) TICAM1; (d) PDGFA; (e) GATA4. ^∗^*P* < 0.05; ^∗∗^*P* < 0.01; ^∗∗∗^*P* < 0.001.

Correlation analysis between 5 IRGs associated with tumor progression and 6 types of immune cells in EC was conducted using the online tool TIMER. The expression of ITPR3 and SLPI had a positive and negative correlation with tumor purity, respectively (Figures 8(a) and 8(b)). Moreover, the expression of SLPI and TICAM1 showed negative correlations with B cell and macrophage infiltrations (Figures 8(b) and 8(c)). We also observed a negative correlation between the SLPI expression and CD4^+^ T cell infiltration and a positive correlation between the TICAM1 expression and dendritic cell (DC) infiltration (Figures 8(b) and 8(c)). Unlike SLPI and TICAM1, PDGFA was positively associated with the proportion of macrophages (Figure 8(d)). Apart from B cells, GATA4 expression exhibited a positive correlation with the infiltration of CD8^+^ T cells and a negative correlation with dendritic cell infiltration, respectively (Figure 8(e)).

**Figure 9:**
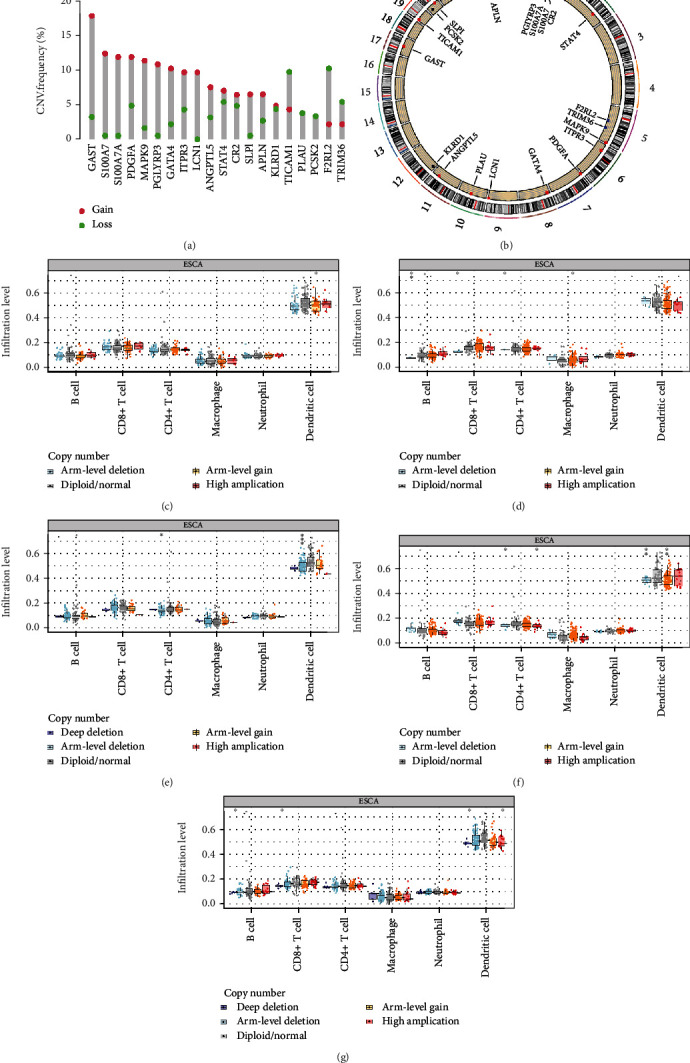
Copy number variations of IRGs. (a, b) Copy number variation and chromosomal position of IRGs in the signature. (c–g) Effect of copy number variation of five key IRGs on immune cell infiltration; (c) ITPR3; (d) SLPI; (e) TICAM1; (f) PDGFA; (g) GATA4. ^∗^*P* < 0.05; ^∗∗^*P* < 0.01; ^∗∗∗^*P* < 0.001.

In addition, we investigated the effect of copy number variation in these progression-related IRGs on immune cell infiltration. CNV information was available for 20 of these 21 prognostic IRGs (Figure 9(a)). The circus plot shows the chromosomal localization and CNV of these IRGs (Figure 9(b)). We found that copies of 4 key IRGs (PDGFA, GATA4, ITPR3, and SLPI) were predominantly increased, while the copy number deletion of TICAM1 was more significant (Figure 9(a)). The arm-level gain copies of ITPR3 could influence the infiltration of dendritic cells (Figure 9(c)). The CNV of SLPI could influence the infiltration of B cells, CD8^+^ T cells, CD4^+^ T cells, and macrophages (Figure 9(d)). The arm-level CNV of TICAM1 and PDGFA could significantly influence dendritic cell and CD4^+^ T cell infiltration (Figures 9(e) and 9(f)). The deep deletion of GATA4 copies could affect B cell, CD8^+^ T cell, and dendritic cell infiltration (Figure 9(g)).

### 3.7. Therapeutic Response Prediction

**Figure 10:**
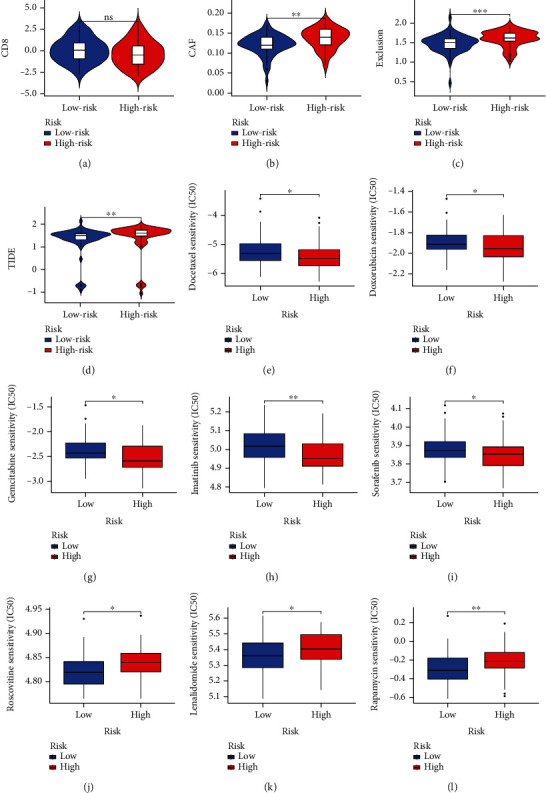
Therapeutic sensitivity analyses. TIDE results revealed differences in CD8^+^ T cell scores (a), CAF scores (b), T cell exclusion scores (c), and TIDE scores (d) between the two groups. High-risk patients sustained a better response to docetaxel (e), doxorubicin (f), gemcitabine (g), imatinib (h), and sorafenib (i). Low-risk patients experienced better responses to roscovitine (j), lenalidomide (k), and rapamycin (l). ^∗^*P* < 0.05; ^∗∗^*P* < 0.01; ^∗∗∗^*P* < 0.001.

To assess the clinical application of this signature, we further evaluated the differential response to immunotherapy and chemotherapy in patients with different risks. High-risk patients had higher CAF (Cancer-associated fibroblast) scores, immune exclusion scores, and TIDE scores, suggesting that they were less likely to benefit from immunotherapy than low-risk patients (Figures 10(b)–10(d)). However, there was no difference in CD8^+^ T cell scores between the two groups, which is consistent to some extent with the results of CIBERSORT (Figure 10(a)). The results of chemotherapeutic effect prediction showed that high-risk patients had lower IC50 scores for docetaxel, doxorubicin, gemcitabine, imatinib, and sorafenib, meaning they were more sensitive to them (Figures 10(e)–10(i)). In contrast, the low-risk group showed higher sensitivity to roscovitine, lenalidomide, and rapamycin (Figures 10(j)–10(l)). These data suggested that high-risk patients exhibited poorer overall survival outcomes and derived limited benefit from immunotherapy. Interestingly, this patient population was more likely to benefit from conventional chemotherapeutic agents such as docetaxel, doxorubicin, and gemcitabine.

## 4. Discussion

Esophageal cancer is a common gastrointestinal tumor with a high degree of malignancy. Immunotherapy is widely thought to have great clinical potential, given its ability to activate the body's immune response [30]. However, immunotherapy has been associated with a low response rate, with only a small percentage of patients exhibiting good immune response and therapeutic effects [31]. The immune cells, such as tumor-infiltrating macrophages, Treg cells, and cytotoxic T cells, regulate the biological behaviors of the tumor and then influence the effect of immunotherapy [32]. Therefore, it is necessary to develop a reliable prognostic signature emphasizing immune-related genes and explore the immune microenvironment characteristics to provide novel insights for individualized immunotherapy of EC patients.

In this research, we introduced a risk-scoring system based on 21 immune-related genes to predict the prognosis of patients with EC. The differentially expressed IRGs were mainly enriched in GO terms such as receptor-ligand activity, regulation of immune effector process, and KEGG pathways such as cytokine interaction and inflammatory signaling processes, showing that immune-related processes and molecules were active in EC. Forty-eight DEIRGs were associated with patient prognosis, 21 of which (S100A7, S100A7A, LCN1, CR2, STAT4, GAST, ANGPTL5, TRAV39, F2RL2, PGLYRP3, KLRD1, TRIM36, PDGFA, SLPI, PCSK2, APLN, TICAM1, ITPR3, MAPK9, GATA4, and PLAU) were further screened to establish the risk signature. Worse survival outcomes and clinicopathological features were found in high-risk patients, indicating the reliable performance of the prognostic signature. Moreover, this immune signature was an independent prognosis indicator. The adherens junction and TGF-*β* signaling pathways were significantly enriched in the high-risk group. In contrast, B cell-mediated immunity and immune activation pathways were enriched in the low-risk group.

We analyzed the characteristics of the tumor microenvironment in patients with different risks. The fractions of CD4^+^ memory resting T lymphocytes and M2 macrophages were significantly higher in the high-risk patients and positively correlated with the risk scores. Memory CD4^+^ T cells play essential roles in rapid immune responses when reexposure to one specific antigen occurs [33]. Lu et al. reported that the proportion of CD4^+^ memory resting T cells increased in EC tissues compared to normal tissues [34]. According to the prognostic model of EC and head and neck squamous cell carcinoma (HNSCC) established by other researchers, tumors in high-risk patients were characterized by a higher proportion of CD4^+^ memory resting T lymphocytes [35, 36]. Researchers also found that in non-small cell lung cancer, EGFR-mutant and ALK-rearranged tumors responded poorly to anti-PD-1/PD-L1 treatment and exhibited higher infiltrations of CD4^+^ memory resting T cells [37]. An increasing body of evidence suggests that tumor-infiltrating M2 macrophage is a suppressive macrophage phenotype in TME, which could induce angiogenesis, suppress the immune response, and affect the efficacy of immunotherapy [11, 38]. It has also been shown that higher M2 macrophage infiltration is associated with unfavorable survival in EC patients [39]. Accordingly, we hypothesized that low-risk patients could respond better to ICB therapy.

T follicular helper (Tfh) cells may play important roles in supporting B cells, recruiting CD8^+^ T cells and natural killing (NK) to facilitate antitumor immunity [40]. Current evidence suggests that Tfh cells produce IL-21, crucial for B cell activation and tumor-infiltrating CD8^+^ T cell effector function, enhancing antitumor immunity and ICB response [41, 42]. A higher percentage of follicular helper T lymphocytes were present in normal esophagus tissues compared to ESCC tissues [34]. Tumor-infiltrating plasma cells in esophageal cancer correlated with positive regulation of adaptive immunity, antitumor activity, and favorable survival [43]. In this study, we found that the proportion of Tfh cells and plasma cells negatively correlated with risk scores, suggesting that low-risk patients with a higher abundance of tumor-infiltrating Tfh cells may be more responsive to ICB therapy.

High-risk patients showed higher expression of TNFSF4 and CD44 and lower expression of CD40 and PDCD1. A negative correlation between risk scores and PDCD1 expression was observed. These four molecules have been reported as targets for immunotherapy [6, 44, 45]. TNFSF4, a member of the tumor necrosis factor ligand family, also known as OX40L, was found to be closely associated with antitumor immunity [46]. Its combination with OX40 could regulate T cell proliferation, activation, and cytokine production [47]. Overwhelming evidence indicates that CD44 could regulate tumor biological characteristics such as initiation, metastasis, and drug resistance [48]. Through downregulation of the Fas-FasL pathway via CD44, cancer cells can reportedly escape the killing of cytotoxic T lymphocytes (CTLs) [49]. Furthermore, it has been shown that CD44^+^ tumor-infiltrating cells could selectively express PD-L1 to evade immune surveillance compared with CD44^−^ cells [50]. CD40, one of the most important stimulatory immune checkpoints, plays an essential role in activating innate and adaptive immune responses [51]. Using CD40 agonists to enhance the CD40-mediated stimulatory signal, activate APCs and other immune cells, and then enhance antitumor immunity has been proven effective against different malignancies [52–55]. In this study, increased expression of CD44 and TNFSF4 and decreased expression of CD40 and PDCD1 were found in patients with higher risk scores.

After analysis of IRG expression characteristics in patients with different clinical features, we mainly focused on 5 IRGs (ITPR3, PDGFA, SLPI, TICAM1, and GATA4) that were associated with the grade, stage, and lymph node metastasis of the tumor, implying that they may exert important roles in EC progression. Downregulation of IP3R3 (inositol 1,4,5-trisphosphate receptor type 3) has been proposed to be oncogenic by promoting proapoptotic mitochondrial Ca2^+^ transfer in breast and prostate cancer [56, 57]. Moreover, the high ITPR3 expression in lung cancer was associated with a better prognosis [58]. In our study, ITPR3 expression declined with a more advanced tumor grade and stage. A positive correlation of ITPR3 expression with tumor purity was observed.

SLPI, known as a secretory leukocyte protease inhibitor, is a serine protease inhibitor and its biological functions include inducing cell proliferation/differentiation and anti-inflammatory, antiviral, and antibacterial functions. There is a rich literature available substantiating that SLPI is overexpressed in diverse cancers, including gastric cancer, ovarian cancer, and pancreatic cancer [59–61]. However, SLPI exhibits low expression in head and neck squamous cell carcinoma compared to other carcinomas and is associated with a better prognosis [62]. In this study, moderate or early-stage tumors exhibited higher expression of SLPI, similar to the results found in HNSCC. Interestingly, researchers found that genetically modified tumor cells with SLPI overexpressing did not exhibit tumorigenesis in immunocompetent mice and may act as a vaccine that partially restrains tumor growth and stimulate the adaptive immune response [63].

GATA4 is a zinc finger transcription factor that belongs to the GATA family. It can regulate specific gene transcription upon binding to GATA elements. Previous studies indicated that GATA4 might act as a putative tumor suppressor gene. Growing evidence suggests the presence of methylation in GATA4 gene promoter regions in gastric, esophageal, and ovarian cancers [64–66]. Interestingly, GATA4 overexpression showed antitumor effects, with inhibited colorectal cancer cell proliferation, migration, and invasion in vitro [67]. In our study, higher expression of GATA4 was observed in early-stage EC. Moreover, the expression of GATA4 positively correlated with CD8^+^ T cell and B cell infiltrations in EC.

Platelet-derived growth factor subunit A (PDGFA), a member of the platelet-derived growth factor (PDGFs) family, plays an essential role in regulating angiogenesis, cell proliferation, migration, and differentiation by binding to PDGF*α*- or *β*-receptors [68]. Moreover, high expression of PDGFs has been associated with poor prognosis and tumor progression in oral squamous cell carcinoma, liver cancer, and colorectal cancer [69–71]. Han et al. reported that a high level of PDGFA was strongly associated with advanced T stage and poor survival of EC patients, consistent with our findings [72]. In addition, our study showed a positive correlation between PDGFA expression and tumor-infiltrating macrophages.

TICAM1 (Toll-interleukin 1 receptor domain (TIR)-containing adaptor molecule) is an adaptor molecule in TLR3-dependent induction of interferon-*β*. TICAM1 and its signaling pathway involve biological processes such as activating antitumor NK (natural killer cell), CTL induction, and DC maturation [73]. The TLR3/TICAM1 pathway has been reported to inhibit polyposis by suppressing the c-Myc expression, leading to longer mice survival [74]. In the present study, we found that the expression of TICAM1 in advanced tumors was lower than in low-grade tumors. The expression of TICAM1 was negatively correlated with tumor-infiltrating macrophages and B cells and positively correlated with DC infiltration. The TLR3-TICAM1-IRF3-IFN-*β* axis activated by a TLR3-specific agonist in DCs has been reported to participate in CD8^+^ T cell cross-priming and relieve innate resistance to ICB therapy without cytokine toxicity [75].

The prognostic and functional regulatory value of other IRGs of this signature in esophageal cancer has also been investigated. For example, TRIM36 expression has been reported to correlate with the size, stage, lymph node metastasis, and *β*-catenin expression of esophageal cancer [76]. Overexpression of TRIM36 inhibited ESCC growth and promoted apoptosis [77]. The proproliferative role of F2RL2 in EC has also been reported [78]. Dysregulation of the miR-204-5p/APLN axis was involved in mediating malignant behaviors such as proliferation, invasion, and stemness in EC [79]. Fang et al. found that tumor-derived PLAU facilitated the inflammatory phenotype conversion of CAFs (cancer-associated fibroblasts), while IL-8 secreted by CAFs promoted PLAU expression in tumor cells, forming a loop to promote ESCC progression [80].

Herein, we also explored differences in predictive responses to immunotherapy and chemotherapy between groups. Of note, high-risk patients exhibited a poor prognosis but showed higher sensitivity to chemotherapeutic drugs such as docetaxel, doxorubicin, gemcitabine, imatinib, and sorafenib, meaning that high-risk patients may benefit from conventional chemotherapy. In contrast, according to the TIDE results, low-risk patients responded better to ICB treatments. Taken together, these results allow us to speculate that high-risk patients may be more suitable for conventional chemotherapy regimens, while low-risk patients can respond better to immunotherapy.

In summary, we developed a reliable prognostic signature emphasizing immune-related genes, which could effectively and independently predict the overall survival of EC patients. The clinical characteristics, the proportion of tumor-infiltrating immune cells, immune checkpoint expression, and sensitivity to chemotherapy and immunotherapy varied between different risk groups. Additionally, five IRGs (ITPR3, SLPI, GATA4, TICAM1, and PDGFA) associated with clinical features and immune cell infiltrations were identified, suggesting their potential as therapeutic targets. However, there are also limitations in our study. Firstly, this was a nonexperimental study that relied heavily on bioinformatics, thus its conclusions need to be validated by subsequent experiments. In addition, the mechanisms by which these IRGs affect the tumor microenvironment and antitumor immunity are still not fully clarified and need further elucidation.

## Figures and Tables

**Table 1:** Prognosis-related IRGs.

ID	Full name	HR	*P* value
S100A7	S100 calcium binding protein A7	0.906290	0.020418
S100A7A	S100 calcium binding protein A7A	0.918288	0.048764
LCN1	Lipocalin 1	1.594471	0.006260
CR2	Complement C3d receptor 2	0.809305	0.025273
STAT4	Signal transducer and activator of transcription 4	0.637901	0.006343
GAST	Gastrin	0.795518	0.048965
ANGPTL5	Angiopoietin like 5	1.510337	0.029443
TRAV39	T cell receptor alpha variable 39	0.548446	0.001660
F2RL2	Coagulation factor II thrombin receptor like 2	1.513455	0.000262
PGLYRP3	Peptidoglycan recognition protein 3	0.834100	0.004133
KLRD1	Killer cell lectin like receptor D1	0.505434	0.005050
TRIM36	Tripartite motif containing 36	0.658894	0.033293
PDGFA	Platelet derived growth factor subunit A	1.387502	0.048138
SLPI	Secretory leukocyte peptidase inhibitor	0.835041	0.012096
PCSK2	Proprotein convertase subtilisin/kexin type 2	1.297815	0.034134
APLN	Apelin	1.264952	0.043527
TICAM1	TIR domain containing adaptor molecule 1	0.621796	0.010537
ITPR3	Inositol 1,4,5-trisphosphate receptor type 3	0.560138	0.004937
MAPK9	Mitogen-activated protein kinase 9	3.783973	0.014732
GATA4	GATA binding protein 4	0.839807	0.047590
PLAU	Plasminogen activator, urokinase	1.529887	0.003680

## Data Availability

The public data could be downloaded at (https://portal.gdc.cancer.gov/ and https://www.ncbi.nlm.nih.gov/geo/).
